# A Jornada para o Controle da Hipertensão Arterial no Brasil

**DOI:** 10.36660/abc.20230533

**Published:** 2023-08-28

**Authors:** Carlos Henrique Miranda

**Affiliations:** 1 Universidade de São Paulo Faculdade de Medicina de Ribeirão Preto Departamento de Clínica Médica Ribeirão Preto SP Brasil Departamento de Clínica Médica, Faculdade de Medicina de Ribeirão Preto, Universidade de São Paulo, Ribeirão Preto, SP – Brasil

**Keywords:** Hipertensão/epidemiologia, Hipertensão/prevenção e controle, Fatores de Risco, Indicadores de Qualidade em Assistência à Saúde/ética, Cooperação e Adesão ao Tratamento

A hipertensão arterial (HA) é um fator de risco cardiovascular, cerebrovascular e renal altamente prevalente em todo o mundo. Esse risco associado pode ser reduzido por meio da instituição de uma terapia bem estabelecida e validada cientificamente. Por essas razões, o controle da HA desempenha um papel na prevenção de doenças crônico-degenerativas na saúde pública.^1^ A taxa de HA controlada reflete a qualidade da assistência à saúde oferecida à população.

O Registro Nacional do Controle da Hipertensão Arterial Avaliado pela Medida de Consultório e Residencial no Brasil: Registro LHAR, recentemente publicado nos Arquivos Brasileiros de Cardiologia, mostra um panorama desse cenário no Brasil.^2^ Alguns achados deste estudo merecem destaque.

Primeiro, uma taxa de HA controlada de 46,4% foi observada usando a medida casual da pressão arterial (PA) no consultório e a monitorização residencial da PA em casa (MRPA). Houve um bom progresso no controle desse fator de risco crítico no Brasil recentemente. Investigações anteriores mostraram uma taxa de HA controlada entre 10,4% e 35,2%.^3^ O primeiro registro brasileiro realizado em 2018, que considerou valores abaixo de 130 × 80 mmHg como controle adequado da PA, mostrou uma taxa de HA controlada de 24,3% no início do estudo e 24,7% após um ano.^4^ O registro atual, considerando essa mesma meta de PA na MRPA, conforme recomendado nas últimas Diretrizes Brasileiras de Hipertensão,^5^ apresentou taxa de HA controlada de 34,9%.

Em segundo lugar, a importância de incorporar MPRA no manejo adequado da HA. Modificou completamente a estratégia terapêutica em um quarto dos pacientes (25%), identificando uma proporção de 15% de hipertensão do avental branco não controlada e 10% de hipertensão mascarada não controlada.

Terceiro, apesar de estudos científicos mostrarem benefícios no tratamento da HA em idosos,^6,7^ parcela da população cada vez mais numerosa em nosso país, a pior taxa de HA controlada (38,1%) foi observada justamente nessa faixa etária que correspondia ao quarto quartil (70-98 anos), índice 10% inferior a outros quartis de idade.

Um aspecto crucial a ser observado na análise de registros desse tipo é a amostragem da população a ser estudada. Apesar de todo o cuidado dos pesquisadores neste registro, a ocorrência de algum viés não pode ser excluída com segurança.

Pacientes hipertensos, principalmente os de classes sociais mais altas, têm fácil acesso ao equipamento do esfigmomanômetro automático. Aqueles pacientes com níveis de pressão arterial mais controlados podem ter maior probabilidade de participar do estudo do que aqueles com níveis de pressão arterial mais elevados. A documentação do número de pacientes triados que não concordaram em participar do estudo ajudaria a dimensionar esse problema.

Existe uma estreita associação entre o nível socioeconômico dos pacientes estudados e as taxas de controle dos fatores de risco cardiovasculares, incluindo a HA, conforme reconhecido pelos autores deste registro. Uma taxa de HA controlada mais alta foi documentada em países desenvolvidos (28,4%) em comparação com países menos desenvolvidos (7,7%).^8^ A amostra do Registro LHAR foi composta por pacientes de 231 centros privados de atendimento especializado em cardiologia. Embora a classe socioeconômica desses pacientes não tenha sido categorizada no registro, o fato é que a assistência médica privada nos leva a uma classe social com maior poder aquisitivo. Mesmo com avanços no acesso à medicação anti-hipertensiva no sistema público de saúde nas últimas décadas no Brasil, por meio do Programa Farmácia Popular do Ministério da Saúde,^9^ consideramos que a possibilidade de taxa de HA controlada em pacientes usuários do Sistema Único de Saúde (SUS) brasileiro seja menor do que a encontrada neste registro.

Um lado positivo da acessibilidade aos esfigmomanômetros automáticos é o uso generalizado da MRPA no tratamento da HA. Porém, vale ressaltar que para o pleno aproveitamento desse método é necessário o uso de equipamentos certificados dentro de um protocolo validado, como o recomendado pela atual Diretriz Brasileira de Hipertensão.^5^

Outro ponto a ser observado é que os pacientes foram incluídos neste registro entre junho e dezembro de 2019, período imediatamente anterior à pandemia de COVID-19. Durante e após essa pandemia, houve uma piora no controle das doenças crônicas em todo o mundo, inclusive no Brasil.^10^

Concluindo, apesar dos avanços observados no controle da HA no Brasil, ainda temos uma longa jornada. Mais da metade dos hipertensos diagnosticados apresentam HA não controlada, e ainda não podemos esquecer daqueles que ainda não tiveram sua HA diagnosticada.
