# Supplementary material for: A Jornada para o Controle da Hipertensão Arterial no Brasil
Source: Arq Bras Cardiol. 2023 Aug 28;120(8):e20230533. [Article in Portuguese] doi: 10.36660/abc.20230533 (PMC10519250; doi:10.36660/abc.20230533)
Supplement: Translation

# Translation: The Journey Towards Hypertension Control in Brazil

**Authors:** Carlos Henrique Miranda

**Affiliations:** 1 Ribeirão Preto SP Brazil Departamento de Clínica Médica, Faculdade de Medicina de Ribeirão Preto, Universidade de São Paulo, Ribeirão Preto, SP – Brazil

**Keywords:** Hypertension/epidemiology, Hypertension/prevention and control, Risk Factors, Health Care Quality Indicators/ethics, Treatment Adherence and Compliance

Arterial hypertension (AH) is a highly prevalent cardiovascular, cerebrovascular, and renal risk factor worldwide. This associated risk can be reduced through the institution of a well-established and scientifically validated therapy. For these reasons, AH control plays a role in preventing chronic degenerative diseases in public health.^1^ The controlled AH rate reflects the quality of health care offered to the population.

The *Arterial Hypertension control rate evaluated by the office and residential measurements: LHAR National Registry*, recently published in the *Arquivos Brasileiros de Cardiologia*, shows an overview of this scenario in Brazil.^2^ Some findings of this study deserve to be highlighted.

First, a controlled AH rate of 46.4% was observed using casual blood pressure (BP) measurement at the office and home BP measurement (HBPM). There has been good progress in controlling this critical risk factor in Brazil recently. Previous investigations showed a controlled AH rate between 10.4% and 35.2%.^3^ The first Brazilian registry conducted in 2018, which considered values below 130 × 80 mmHg as adequate BP control, showed a controlled AH rate of 24.3% at baseline and 24.7% after one year.^4^ The current registry, considering this same BP goal in HBPM, as recommended in the last Brazilian Guidelines of Hypertension,^5^ showed a controlled AH rate of 34.9%.

Second, the importance of incorporating HBPM in proper AH management. It completely modified the therapeutic strategy in a quarter of the patients (25%), identifying a proportion of 15% of uncontrolled white coat hypertension and 10% of uncontrolled masked hypertension.

Third, despite scientific studies showing benefits in the treatment of AH in older people,^6,7^ a portion of the population that is increasingly numerous in our country, the worst controlled AH rate (38.1%) was observed precisely in this age group that corresponded to the fourth quartile (70-98 years), an index 10% lower than other age quartiles.

A crucial aspect to be observed while analyzing records of this type is sampling the population to be studied. Despite all the care taken by the researchers in this registry, the occurrence of some bias cannot be safely excluded.

Hypertensive patients, especially those from higher social classes, can easily access the automatic sphygmomanometer equipment. Those patients with more controlled blood pressure levels could be more likely to participate in the study than those with higher blood pressure levels. Documentation of the number of screened patients who did not agree to participate in the study would help to dimension this problem.

There is a close association between the socioeconomic level of the studied patients and the rates of cardiovascular risk factors control, including AH, as recognized by the authors of this registry. A higher controlled AH rate has been documented in developed countries (28.4%) compared to less developed countries (7.7%).^8^ The LHAR Registry sample consisted of patients from 231 private centers of specialized care in cardiology. Although the socioeconomic class of these patients was not categorized in the registry, the fact that private medical care takes us to a social class with greater purchasing power. Even with advances in access to antihypertensive medication in the public health system in recent decades in Brazil, through the Farmácia Popular Program of the Ministry of Health,^9^ we consider the possibility of controlled AH rate in patients using the Brazilian Unified Health System (SUS) is lower than that found in this registry.

A positive side of the accessibility to automatic sphygmomanometers is the widespread use of HBPM in AH management. However, it is worth emphasizing that for the full benefit of this method, it is necessary to use certified equipment within a validated protocol, such as the one recommended by the current Brazilian Guideline of Hypertension.^5^

Another point to note is that patients were included in this registry between June and December 2019, a period immediately before the COVID-19 pandemic. During and after this pandemic, there was a worsening in chronic disease control worldwide, including in Brazil.^10^

In conclusion, despite the progress observed in AH control in Brazil, we still have a long journey. More than half of the diagnosed hypertensive patients have uncontrolled AH, and we still cannot forget those who have not yet had their AH diagnosed.
